# Fibrillary glomerulonephritis: a rare entity with unique ultrastructural characteristics

**DOI:** 10.1590/2175-8239-JBN-2022-0004en

**Published:** 2022-07-25

**Authors:** Vlatko Karanfilovski, Galina Severova, Pavlina Dzekova-Vidimliski

**Affiliations:** 1 University Hospital of Nephrology, Skopje, R of North Macedonia

**Figure 1:**
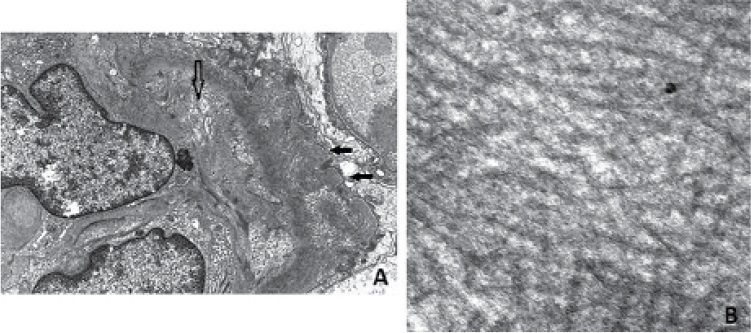
Electron microscopy evaluation demonstrates deposition of organized fibrils with consecutive expansion of mesangial matrix and permeation of lamina densa of the glomerular basal membrane (GBM) (Image A: hollow arrow). Visceral epithelial cells (podocytes) show complete effacement of foot process (Image A: black arrows). At higher magnification (x 60.000), the fibrils are randomly arranged and non-branching, measuring 15 to 18 nm in diameter (Image B).

Fibrillary glomerulonephritis (FGn) is characterized by deposition of randomly arranged polyclonal immune deposits in glomerular matrix^1,2^. A 56-year-old hypertensive patient presented to our hospital with proteinuria (3.04 g/24 hours) and an elevated serum level of creatinine (391 µmol/L). Electron microscopic evaluation of kidney biopsy specimens set the diagnosis of fibrillary glomerulonephritis (Figure 1). There was no evidence of monoclonal components in the blood and urine. Antinuclear and anti-double-stranded DNA antibodies, complement components C_3_ and C_4,_ and markers of viral hepatitis were also negative. The benefit of immunosuppressants is limited,^3^ and half of patients progress to kidney failure within 2 years^1-3^.
